# Role of Oxidative Stress and Nrf2/KEAP1 Signaling in Colorectal Cancer: Mechanisms and Therapeutic Perspectives with Phytochemicals

**DOI:** 10.3390/antiox10050743

**Published:** 2021-05-07

**Authors:** Da-Young Lee, Moon-Young Song, Eun-Hee Kim

**Affiliations:** College of Pharmacy and Institute of Pharmaceutical Sciences, CHA University, Seongnam 13488, Korea; angela8804@naver.com (D.-Y.L.); wso219@naver.com (M.-Y.S.)

**Keywords:** colorectal cancer, oxidative stress, ROS, lipid peroxidation, Nrf2, KEAP1, antioxidant enzymes, phytochemical

## Abstract

Colorectal cancer still has a high incidence and mortality rate, according to a report from the American Cancer Society. Colorectal cancer has a high prevalence in patients with inflammatory bowel disease. Oxidative stress, including reactive oxygen species (ROS) and lipid peroxidation, has been known to cause inflammatory diseases and malignant disorders. In particular, the nuclear factor erythroid 2-related factor 2 (Nrf2)/Kelch-like ECH-related protein 1 (KEAP1) pathway is well known to protect cells from oxidative stress and inflammation. Nrf2 was first found in the homolog of the hematopoietic transcription factor p45 NF-E2, and the transcription factor Nrf2 is a member of the Cap ‘N’ Collar family. KEAP1 is well known as a negative regulator that rapidly degrades Nrf2 through the proteasome system. A range of evidence has shown that consumption of phytochemicals has a preventive or inhibitory effect on cancer progression or proliferation, depending on the stage of colorectal cancer. Therefore, the discovery of phytochemicals regulating the Nrf2/KEAP1 axis and verification of their efficacy have attracted scientific attention. In this review, we summarize the role of oxidative stress and the Nrf2/KEAP1 signaling pathway in colorectal cancer, and the possible utility of phytochemicals with respect to the regulation of the Nrf2/KEAP1 axis in colorectal cancer.

## 1. Introduction

According to the 2020 American Cancer Society report, colorectal cancer was the third highest in both incidence and mortality [1]. Chronic inflammation has been known to be an important factor in the progression of cancer [2]. As proof of this, there is a high prevalence of colorectal cancer (CRC) in inflammatory bowel disease (IBD) patients, such as those with Crohn’s disease (CD) and ulcerative colitis (UC), which is classified as colitis-associated colorectal cancer (CAC) [3]. Recently, reactive oxygen species (ROS) have been studied as a major signaling molecule that plays an important role in the progression of inflammatory diseases [4]. ROS are known as metabolites with strong oxidative capacity and are involved in several intracellular signal transductions [4]. ROS are known to disrupt double-stranded DNA, deform purines, pyrimidines, or deoxyribose, and induce transcriptional repression or induction, replication, and error, all of which are associated with carcinogenesis [5,6]. In addition to ROS, lipid peroxidation has also been shown to be associated with cancer [7]. Lipid peroxidation means that ROS attacks membrane phospholipids containing polyunsaturated fatty acids (PUFA) [7]. It has been found that the by-products produced in this process can regulate cellular signaling pathways by acting as a second messenger of oxidative stress due to their long half-life and diffusion ability, unlike ROS, with their short half-life [8]. For this reason, research on the mechanism and role of ROS and lipid peroxidation has been actively conducted as it has been found to be related to various diseases and carcinogenesis [7,9]. The nuclear factor erythroid 2-related factor 2 (Nrf2)/Kelch-like ECH-associated protein 1 (KEAP1) pathway is known to protect cells from oxidative stress and inflammation, as well as being a major regulator of cellular protective responses to endogenous and exogenous stresses caused by ROS and electrophiles [10,11]. In particular, Nrf2, a well-known factor that regulates ROS, has been shown to be involved in the regulation of IBD by enabling redox regulation and suppressing inflammation and tissue damage [11,12,13]. Based on this, ROS’ scavenging effect on phytochemicals and the ability to remove carcinogens through the antioxidant effect of Nrf2 are attracting attention [14]. In this review, we will discuss the mechanisms by which oxidative stress contributes to colon carcinogenesis, the antioxidant effects mediated by the Nrf2/KEAP1 signaling pathway, and the efficacy of phytochemicals in CRC through the Nrf2/KEAP1 pathway.

## 2. Oxidative Stress and Lipid Peroxidation in Colon Carcinogenesis

**Figure 1:**
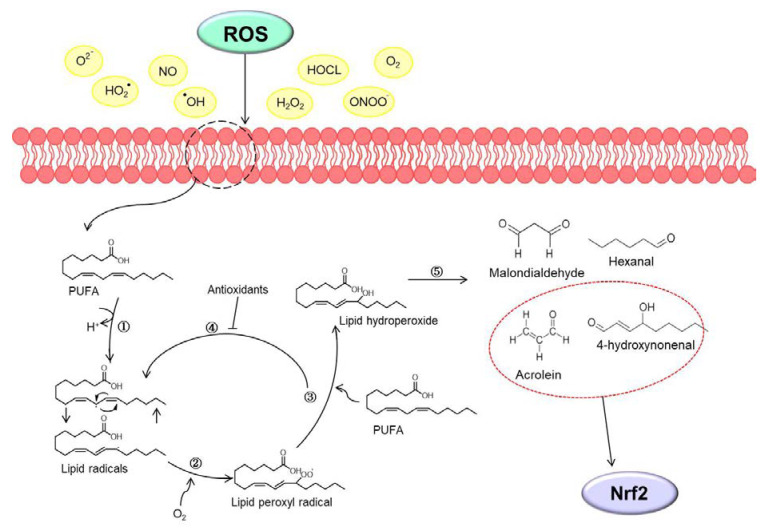
Schematic image of the lipid peroxidation. (1) Initiation phase: reactive oxygen species (ROS) interact with polyunsaturated fatty acids (PUFA), which loses one hydrogen ion and transforms lipid radicals. (2) Propagation phase: lipid radical reacts with oxygen to form (3) lipid hydroperoxide. (4) Termination phase: antioxidants inhibit the propagation stage by donating a hydrogen atom to lipid peroxyl radicals. (5) Finally, lipid hydroperoxide degrades into second oxidative stress messengers such as malondialdehyde, hexanal, 4-hydroxynonenal and acrolein. In particular, 4-hydroxynonenal and acrolein of reactive secondary products interact with the nuclear factor erythroid 2-related factor 2 (Nrf2).

Oxidative stress means an imbalance in redox homeostasis. The cause of the redox imbalance is known as oxidized glutathione or an abnormal NADPH/NADP^+^ ratio. ROS are known to be a major source of oxidative stress. ROS are mainly produced as by-products of intracellular mitochondria and contain all free radicals, such as superoxide anions (O_2_^•−^), perhydroxyl radical (HO_2_^•^), hydroxyl radicals (^•^OH), nitric oxide (NO), hydrogen peroxide (H_2_O_2_), oxygen (O_2_), hypochlorous acid (HOCL), and peroxynitrite (ONOO^−^) [15]. In particular, the production of ROS in biological membranes is very high due to molecular oxygen solubility, so membrane phospholipids, including PUFA, are defenseless against ROS [16]. In this state, the process by which oxidizing agents such as free radicals or non-radical species attack PUFA is lipid peroxidation [17]. When lipid peroxidation begins, it takes place in a chain reaction until the end product is produced. Reactive intermediates produced by oxidative stress cause lipid peroxidation of PUFA to form lipoperocyl radicals and react with lipids and with lipid hydrogen peroxide. Lipid hydrogen peroxide again generates new peroxyl and alkoxy radicals and breaks down into secondary products [17]. Free radicals produced during lipid peroxidation have a short lifespan, but decomposition products from lipid peroxides such as malondialdehyde, hexanal, 4-hydroxynonenal, and acrolein can act as second messengers of oxidative stress due to their long half-life and diffusion capacity [8]. This section discusses 4-hydroxynonenal and acrolein, the secondary oxidative stress messengers involved in the Nrf2/KEAP1 pathway. (Summarized in Figure 1).

### 2.1. 4-Hydroxynonenal

Firstly, 4-hydroxynonenal is the end product produced from the oxidation of n-6 PUFAs [18]; 4-hydroxynonenal is a substance that interacts with proteins, lipids containing amino groups, and nucleic acids, and can regulate many biological functions [19]. In particular, 4-hydroxynonenal is involved in the activation of Nrf2 and upregulates the expression of genes such as heme oxygenase-1 (HO-1), glutathione S-transferase (GST), NAD(P)H quinone oxidoreductase 1 (NQO1), or glutamate-cysteine ligase (GCL), thereby performing cellular antioxidant defense and oxidative stress regulation [20]. In addition, 4-hydroxynonenal induces translocation of Nrf2 to the nucleus, and Nrf2 that has moved to the nucleus recognizes the electrophile response element (EpRE) sequence and forms a heterodimer with c-jun to activate the expression of antioxidant genes [21]. Nrf2 is the primary sensor of oxidative stress, and the absence of Nrf2 induces the accumulation of 4-hydroxynonenal by removing the antioxidant signal [22]. Induction of strong oxidative stress due to 4-hydroxynonenal formation in advanced cancer cells can be a therapeutic strategy to induce apoptosis [23]. Although high levels of 4-hydroxynonenal are known to have many associations with diseases, the levels of lipid peroxidation products in cancer cells are still debatable [18]. 

In the colon, intestinal cells are the main targets of 4-hydroxynonenal because they exist in the lumen and at the interface and can be directly exposed to high concentrations of 4-hydroxynonenal [23]. Additionally, 4-hydroxynonenal can be produced by macrophages infected with gut microbiota, which can be toxic to colon cells [23]. Because 4-hydroxynonenal is highly diffusible, it is known that it can also act as a paracrine signaling molecule [24]. When colon cancer progresses, 4-hydroxynonenal is produced in the lumen of the colon. Normal cells are sensitive to 4-hydroxynonenal, but tumor cells have been shown to be resistant to 4-hydroxynonenal, which can ultimately lead to the development of CRC [25,26]. In fact, 4-hydroxynonenal has been reported to be associated with a decrease in transforming growth factor-βI (TGF-βI), which is known to be downregulated in human colon cancer [27]. Since a decrease in TGF-βI is associated with carcinogenesis, downregulation of 4-hydroxynonenal also suggests that it is related to carcinogenesis [27]. In addition, adenomatous polyposis coli gene mutations have been shown to provide benefits to colorectal cancer cells by promoting cancer growth through effective detoxification of 4-hydroxynonenal [25]. Moreover, heme iron present in myoglobin or hemoglobin induces the oxidation of fats to form toxic lipid peroxidation, such as malondialdehyde and 4-hydroxynonenal [28], which is considered to promote colon carcinogenesis by increasing cell proliferation in the colonic mucosa. These findings imply that cancer cells can provide a selective advantage over normal cells by enhancing their metabolic capacity to 4-hydroxynonenal due to the persistent Nrf2/KEAP1/EpRE pathway. Therefore, further studies on the effect of 4-hydroxynonenal on cells representing various stages of cancer, from normal cells to pre-tumor and neoplasms, are needed (detailed in Table 1).

### 2.2. Acrolein

Acrolein is originally produced from the glycerol component of triacyl and diacyl glycerides, but many studies have reported that acrolein is produced from fatty acids due to oxidative decomposition [29]. In fact, a small amount of acrolein was detected as PUFA was oxidized, but there are reports that it is difficult to detect the amount of acrolein in monounsaturated fatty acids, oleic acid, or ethyl esters [30]. However, it has been reported that it may be produced by auto-oxidation in the ethyl ester of n-3 PUFA, all-cis-7, 10, 13, 16, 19-docosapentaenoic acid [31]. Acrolein is around 10 to 1,000 times more toxic than formaldehyde or 4-hydroxynonenal and is mainly present in cigarette smoke [32]. Since acrolein has a strong electrophilic reactivity to nucleophiles, it interferes with the redox regulation of protein functions and causes cytotoxicity through irreversible reactions [33]. In addition, acrolein has been found to cause several diseases [33]. It has been reported that acrolein upregulates phase II enzymes by regulating the transcriptional activity of genes derived from the ARE promoter and induces cell protection against electrophilic stress [29]. In fact, it was found that the expression of the antioxidant enzyme HO-1 was increased in endothelial cells treated with acrolein [34]. It has also been reported that treatment of acrolein in human lung cancer A549 cells increases the transcription of gamma-glutamylcysteine synthase and normalizes the level of glutathione [35]. In addition, acrolein has been reported to induce the transcription of phase II genes such as NQO-1 by activating Nrf2 [35]. Regarding colon cancer, treatment with acrolein has been reported to increase the levels of acrolein-derived DNA adduct and induce cancer cell death in colon cancer HT29 cells [36]. It was also confirmed that acrolein-induced DNA damages (Acr-dG) were higher in tumor tissues when comparing the cancerous and normal tissues of CRC patients [37]. In addition, myeloperoxidase can generate acrolein, which forms a covalent adduct with the phosphatase tensin homolog tumor suppressor, thereby enhancing the subsequent activation of Akt proto-oncogene signaling [38]. These results suggest that not only acrolein but also myeloperoxidase, an enzyme that can produce acrolein, could be an important factor in determining the risk of colon cancer through endogenous exposure (detailed in Table 1).

**Table 1:** Effects of lipid peroxidation products in colorectal cancer.

Lipid Peroxidation Products	Model	Mode of Action	Reference
4-hydroxynonenal	CaCO-2	4-hydroxynonenal ↓ TGF-βI ↓ Apoptosis ↓	[27]
	APC^+/+^ APC^Min/+^ (colon epithelial cell)	4-hydroxynonenal metabolism ↑ (APC^Min/+^) Aldehyde dehydrogenase 1A1, 2, 3A1 ↑ (APC^Min/+^) Glutathione transferase A4-4 ↑ (APC^Min/+^) Cystine transporter XCT ↑ (APC^Min/+^)	[25]
Acrolein	HT29	Apoptosis ↑ (acrolein 150, 200 μM) DNA adduct ↑	[36]
	CCD-841CoN	p-EGFP ↑ (acrolein 5 μM, 8 h) RAS ↑ (acrolein 5 μM, 8 h) p-AKT ↑ (acrolein 5 μM, 8 h) p-ERK ↑ (acrolein 5 μM, 8 h) Cyclin D1 ↑ (acrolein 5 μM, 8 h)	[37]
	CRC patients	Acr-dG ↑ (in tumor) Over survival (high Acr-dG > low Acr-dG)	[37]
	APC^Min/+^	Cocalent adduct with PTEN ↑ p^ser473^Akt ↑	[38]

TGF-βI; transforming growth factor, APC; adenomatous polyposis coli, Min; multiple intestinal neoplasia, p-EGFR; phospho-epithelial growth factor receptor, p-AKT; phospho-protein kinase B, p-ERK; phospho-extracellular-signal-regulated kinase, Acr-dG; acrolein-induced DNA damages, PTEN; phosphatase and tensin homolog.

## 3. Antioxidative Nrf2/KEAP1 Signaling Pathway

### 3.1. Nrf2

**Figure 2:**
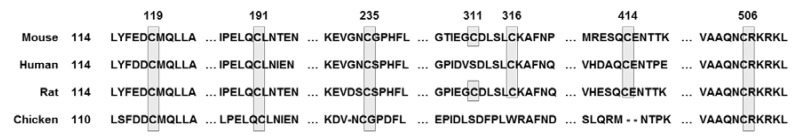
Conserved cysteine residue of Nrf2 across mouse, human, rat, and chicken; cysteine residues shaded and boxed.

**Figure 3:**
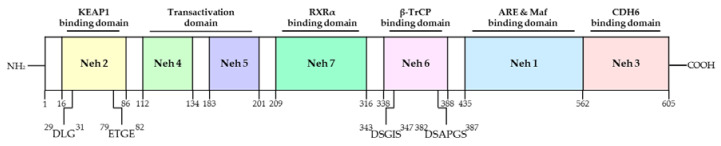
Domain structures and functional domains of Nrf2. The Nrf2 protein contains 7 domains, Neh1-7. The N-terminal Nrf2-ECH homology (Neh) 2 domain has ETGE and DLG motifs that bind to the Kelch domain of Kelch-like ECH-associated protein 1 (KEAP1), resulting in Cullin 3 (Cul3)-dependent E3 ubiquitination and proteasome degradation. The Neh4 and Neh5 domains mediate the transactivation activity of Nrf2. The Neh7 domain inhibits transcription of the Nrf2 target gene. The Neh6 domain has DSGIS and DSAPGS motif. The Neh1 domain helps Nrf2 dimerization with DNA binding to other transcription factors. The C-terminal Neh3 domain supports the Nrf2 transcriptional activity. Adapted from Ooi et al. [57]. NH_2_; N-terminal, COOH; C-terminal, RXRα; retinoed X receptor-alpha, β-TrCP; beta-transducin repeat containing E3 ubiquitin protein ligase, ARE; antioxidant response element, CDH6; chromo-ATPase/helicase DNA-binding protein 6.

Nrf2 was first discovered in the homolog of the hematopoietic transcription factor p45 NF-E2 [39]. The transcription factor Nrf2 is a member of the Cap ‘N’ Collar (CNC) family, which contains conserved basic leucine zipper (bZIP) proteins [40]. To date, six members have been identified in this family, including NF-F2, Nrf1, Nrf2, Nrf3, Bach1, and Bach2 [41]. Several homolog domains of the same Nrf2 gene were identified in human, mouse, chicken, and zebrafish (Figure 2). Seven functional domains from Nrf2-ECH homology (Neh) 1 to Neh7 are known as Nrf2 domains (Figure 3). The N-terminal Neh2 domain of Nrf2 has seven lysine residues that are suitable for ubiquitin conjugation, and the ETGE and DLG motifs are present, so it is bound to the Kelch domain of KEAP1, resulting in Cullin 3 (Cul3)-dependent E3 ubiquitination and proteasome degradation [42,43,44]. The transcriptional activity of Nrf2 has been reported to be improved when the N-terminal Neh2 domain is deleted [45]. The Neh4 and Neh5 domains are rich in acidic residues and have been shown to interact with the CH3 domain of cAMP response element binding protein (CREB)-binding protein (CBP) to mediate the transactivation activity of Nrf2 [46]. The Neh7 domain has been shown to interact with retinoic X receptor alpha as a potential target capable of suppressing CNC-bZIP factors and to suppress transcription of the Nrf2 target gene [47]. The Neh6 domain has two motifs, DSGIS and DSAPGS of β-transducin repeat-containing protein (β-TrCP), and β-TrCP functions as a substrate adapter for the ubiquitin ligase complex [48]. Among them, DSGIS phosphorylation is regulated by glycogen synthase kinase-3 (GSK-3) and is known to enable β-TrCP to ubiquitinate Nrf2 [48]. The Neh1 domain has a CNC-type basic leucine zipper DNA binding motif that helps Nrf2 to dimerize with DNA binding to other transcription factors [45]. Nrf2 cannot bind to the antioxidant/electrophile response element (ARE/EpRE) with a monomer or homodimer, so it must be heterologous to either of the small Maf proteins for DNA binding and transactivation. It was also revealed that the Neh1 domain interacts with the ubiquitin-conjugating enzyme (UbcM2) to regulate the stability of Nrf2 [49]. The C-terminal Neh3 domain has been shown to support Nrf2 transcriptional activity by regulating chromo-ATPase/helicase DNA-binding protein 6 (CHD6), a member of the CHD family known as the Nrf2 transcriptional co-activator [50]. The main function of Nrf2 is to induce various genes that can counter the effects of exogenous and endogenous factors such as oxidative stress and activate the antioxidant response [51]. It is also known that Nrf2 binds Maf to ARE, which is similar to the NF-E2-binding motif, and induces the expression of drug-metabolizing enzymes such as GST and NQO1 [52]. Due to these roles of Nrf2, it has been regarded as a major mechanism of cell defense and a regulator of cell survival [53]. In fact, knockout of Nrf2 in mice increases the susceptibility of mice to diseases associated with oxidative diseases [54]. Talalay et al. have also reported that chemical protective agents can be used to increase the activity of Nrf2 to protect cells against oxidative insults [55]. Recently, activation of Nrf2 has been shown to protect against a variety of diseases, including inflammation and cancer [56].

### 3.2. KEAP1

**Figure 4:**
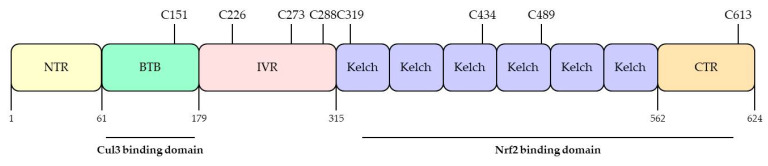
Domain structures and functional domains of KEAP1. The Keap1 protein contains Broad complex/Tramtrack/Bric-a-brac (BTB) domain, the intervening region (IVR) domain, and the Kelch domain. The BTB domain binds to Cul3 and forms homologs and dimers. The IVR domain contains C226, C273, and C288, which regulate Nrf2 activity. The Kelch domain has 6 conserved Kelch repeat sequences in the C-terminal domain, which also interact with the Nrf2. Adapted from Ooi et al. [57]. NTR; N-terminal, CTR; C-terminal, Cul3; cullin-3.

KEAP1 was identified as a negative regulator of Nrf2 in 1999 by Itoh and his colleagues [45]. KEAP1 has three major domains, including the Broad complex/Tramtrack/Bric-a-brac (BTB) domain, the intervening region (IVR) domain, and the Kelch domain, also called the double glycine repeat (DGR) domain (Figure 4). The BTB domain resides at the N-terminal, binds to Cul3, and forms homologs and dimers [58]. The IVR domain connects the BTB domain and the Kelch domain and is rich in cysteine residues essential for KEAP1 activity [44,59]. The Kelch domain has six conserved Kelch repeat sequences in the C-terminal domain [60], which also interacts with the Neh2 domain of Nrf2 and plays an important role in the interaction of KEAP1 and Nrf2 [45]. KEAP1 is a very cysteine-rich protein, with 25 mouse and 27 human cysteine residues [60]. KEAP1 has several cysteine sensors, especially the C151, C273, and C288 cysteine sensors, which have been found to induce Nrf2 into the nucleus and the expression of target genes by modifying the structure of KEAP1 by oxidizing agents and electrophiles [61]. Many plant-derived affinity electronic substances are known to induce a cytoprotective response by activating the Nrf2-KEAP1 axis, in which case the cysteine residue of KEAP1 has been found to act as a sensor [62,63]. Therefore, KEAP1 is considered a biosensor of electrophiles and ROS [64].

### 3.3. Antioxidative Effect by Nrf2/KEAP1 Signal

**Figure 5:**
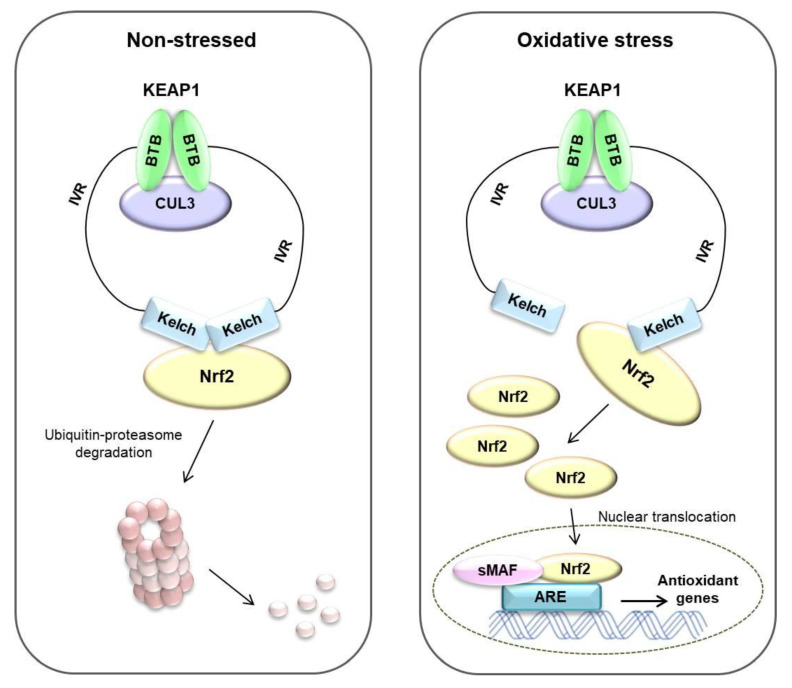
Nrf2/KEAP1 pathway in non-stressed and oxidative stress status. Under non-stressed state, Nrf2 binds to Keap1 by ubiquitin E3 ligase complex. Nrf2 rapidly degrades via ubiquitin–proteasome system. Under oxidative stress state, the ubiquitin E3 ligase activity of the KEAP1-Cul3 complex is reduced, and Nrf2 translocates to the nucleus and is heterologous to the small-MAF (sMAF) protein and binds to ARE. Finally, antioxidant genes are induced by these systems. Adapted from Raghunath et al. [69].

Oxidative stress affects many diseases, including cancer [6]. To overcome these stresses, cells have their own systems to maintain homeostasis [65]. The Nrf2/KEAP1 system is well known as a mechanism for protecting the body from external factors. KEAP1 is an electrophilic sensor, and Nrf2 is an effector for cytoprotective gene activity. In the cytoplasm, KEAP1 forms ubiquitin E3 ligase complex with CUL3 and Nrf2, which rapidly degrades Nrf2 via the proteasome system. Therefore, in the absence of stress, Nrf2 is continuously degraded by KEAP1. However, when exposed to electrophiles or ROS, the cysteine residue of KEAP1 is deformed, the ubiquitin E3 ligase activity of the KEAP1-Cul3 complex is reduced, and Nrf2 is activated. Activated Nrf2 translocates to the nucleus and is heterologous to the sMAF protein and binds to ARE [64]. ARE is located in the promoter region of multiple genes that encode phase II detoxifying enzymes and antioxidant proteins [66]. ARE is crucial for the transcriptional activation of the antioxidant genes such as NQO1, GSTs, glutamate-cysteine ligase, and HO-1 [67,68] (summarized in Figure 5).

### 3.4. Nrf2/KEAP1-Dependent Regulation of Antioxidative Enzymes

#### 3.4.1. Superoxide Dismutase 

Superoxide dismutase (SOD) is the cell’s key antioxidant, making the harmful superoxide anions less dangerous by transforming the two molecules of superoxide anions (O_2_^•−^) into hydrogen peroxide (H_2_O_2_) and oxygen (O_2_) [70]. A number of studies have shown that ROS act as carcinogens by inducing mutations in cells [71]. SOD requires cofactors for its activity as a metal enzyme [72]. In general, the metal ions bound by SOD are iron (Fe), zinc (Zn), copper (Cu), and manganese (Mn) [70]. SOD is classified into three types: Fe-SOD found in prokaryotes and chloroplasts in plants, Mn-SOD in mitochondria in prokaryotes and eukaryotes, and Zn-SOD, which is widely distributed in eukaryotes [73,74]. Interestingly, the activities of Cu-SOD, Mn-SOD, and Zn-SOD have been revealed to decrease in cancer cells [75,76]. SOD protects cells from excess oxygen radicals, free radicals, aging, and other harmful substances [70]. Normalized SOD levels have been reported to help to reverse the phenotype of cancer cells [75]. SOD has been known to regulate the progression of cancer, so it could be used as a new target for cancer treatment [77]. The levels of SOD decrease with aging, while the formation of free radicals increases. It has been suggested that supplementation of SOD would protect the immune system, protect against diseases, and ultimately delay aging [39]. Natural sources of SOD include cabbage, wheat grass, barley grass, and broccoli, and adequate intake of SOD could prevent cancer through antioxidant suppression [78,79].

#### 3.4.2. Catalase 

Catalase is a protein with four subunits [80]. Catalase is a very common antioxidant enzyme and is present in almost every tissue that requires oxygen [70]. Catalase uses iron (Fe) or manganese (Mn) as cofactors to break down or reduce hydrogen peroxide (H_2_O_2_) into water and oxygen (O_2_) [81]. Hydrogen peroxide plays a role in regulating physiological processes such as cell proliferation, cell death, carbohydrate metabolism, mitochondrial function, and platelet activation, but at high concentrations, it is very harmful to cells, so the role of catalase is important [82,83]. However, the levels of Catalase in cancer and normal tissues are still controversial, so further research on it is needed [84].

#### 3.4.3. Glutathione Peroxidase 

Glutathione peroxidase (GPx) is an important enzyme that breaks down hydrogen peroxide (H_2_O_2_) into water in cells [85]. It is also called selenocysteine peroxidase because the activity of GPx depends on the micronutrient selenium. GPx protects cells from oxidative stress by inhibiting the lipid peroxidation process [73]. There are at least eight enzymes present in humans, from GPx1 to GPx8 [86]. Among them, GPx1 is the most abundant and is present in virtually every cell [87]. In addition, GPx2 is mainly found in the gastrointestinal tract of the intestine, and GPx3 is mainly located in the kidneys [88,89]. Several studies have demonstrated the clinical significance of GPx. It has been found that low GPx activity in the body tends to impair antioxidant protection, causing oxidative damage to functional proteins and neurotoxic damage [70,90]. It has also been reported that GPx1 deficiency increases vascular oxidative stress and causes endothelial dysfunction [91]. However, although it has been found that the level of GPx1 is not associated with colorectal cancer, it has been reported that the genotype of GPx1 and lifestyle factors might influence the development of colorectal cancer [92,93].

#### 3.4.4. Glutathione

Glutathione (GSH) plays an important role in eliminating ROS [94]. GSH is endogenously synthesized in the body and is found in almost every cell [95]. GSH plays many roles in antioxidant defense, redox regulation, cysteine storage and transport, and cell proliferation regulation and synthesis [95]. GSH directly or indirectly removes free radicals through enzymatic reactions [96]. In particular, the thiol moiety present in GSH has been found to play a major role in the direct removal of ROS [96]. Other antioxidant enzymes, especially oxides that cannot be removed due to SOD defects, are immediately compensated by the induction of GSH synthesis, protecting cells from oxidative stress and cell death [97]. GSH can utilize quinones that cycle through redox to increase the production of hydrogen peroxide, and by increasing the transcription of glutamate-cysteine ligase catalytic subunit (GCLC), it is possible to maintain the concentration of GSH in cells continuously [94,98]. It also induces the expression of GCLC or GCL modifier subunit (GCLM) by increasing the concentration of nitric oxide or electrophiles that can induce hydrogen peroxide production, which can generate oxidative stress [99]. ARE, an antioxidant response element, exists in the GCLC and GCLM promoters, and one of the established transcription factors capable of binding to ARE is known as Nrf2 [45,100]. Decreased levels of GSH in the body have been shown to exacerbate some diseases [101]. Although increasing GSH synthesis may be useful, natural compounds such as curcumin or sulforaphane, which is present in broccoli, have also been suggested [102,103]. However, since these natural products are limited from being used as main treatments, more effective drug development is needed [94].

#### 3.4.5. Heme Oxygenase-1 

HO-1, 32 kDa in size, is one of the isoforms of heme oxygenase, an enzyme that breaks down heme into free iron, carbon monoxide, and biliverdin [104]. In mammals, it is present at low levels but is rapidly upregulated by several oxidative stimuli, such as ROS, UV, growth factors, and inflammatory cytokines [105]. HO-1 is known to be present predominantly in microsomes but has also been found to be present in the mitochondria or nucleus [106,107,108]. HO-1 plays a major role in preventing ROS production by reducing intracellular Fe^2+^ [104]. HO-1 is known to play a role in regulating gene transcription through migration to the nucleus, thereby promoting cell protection against oxidative stress [108]. The synthesis of HO-1 is mainly regulated at the transcription level [109]. Indeed, various transcription factors activated by oxidative stress, such as activator protein-1, hypoxia-inducible factor-1, nuclear factor kappa-light-chain-enhancer of activated B cells, and Nrf2, bind to the promoter region of HO-1 [110]. Demonstrating the traditional function of HO-1 as an antioxidant enzyme, it has been revealed that levels of oxidized proteins and lipid peroxides, as well as ROS generation, are also elevated in HO-1 knockout mice [111]. However, HO-1 also participates in the development and progression of several types of cancer [112]. Upregulation of HO-1 is known to be associated with cancer growth and resistance to chemotherapy through the induction of angiogenesis and metastasis and inhibition of apoptotic cancer cell death [112,113]. In addition, it has been shown that chemotherapy and anticancer treatments such as radiotherapy increase the expression of HO-1, and HO-1 inhibitors may be sensitive to cancer treatment [114]. Therefore, the induction of HO-1 has the potential to be associated with carcinogenesis under certain conditions, so it must be considered very carefully in clinical application.

#### 3.4.6. NAD(P)H Quinone Oxidoreductase 1 

NQO1 is a flavoenzyme that catalyzes the two- and four-electron reduction of endogenous and exogenous quinones [115]. The reducing action catalyzed by NQO1 is very important for cells to prevent redox circulation, which regulates free radicals [116]. Therefore, NQO1 protects cells from oxidative stress and external factors [117]. NQO1 reduces the quinone sequence to its hydroquinone using the reduced form of the pyridine nucleotide contributor NADH or NADPH as a donor [118]. Expression of NQO1 has been observed in the rat liver and in several tissues in humans, especially colon, pancreas, lung, and breast tumors, and the levels of NQO1 have been reported to be controlled by ARE in an oxidative stress environment [119]. The promoter region of NQO1 contains the ARE sequence, which was found to be regulated by Nrf2 [120]. Nrf2 binds to ARE and consequently induces the expression of NQO1 protein. Indeed, knockout of Nrf2 has been reported to decrease the expression of NQO1 [121]. Although the activity of NQO1 has been implicated in the development of human cancer, the clinical significance of NQO1 has yet to be elucidated [119]. However, strategies to increase the efficacy of bio-reductive anticancer drugs by inducing the activity of NQO1 are in the spotlight [119].

## 4. Regulation of Nrf2/KEAP1 by Phytochemicals in Colorectal Cancer

Various phytochemicals in the diet have anticancer activity [122]. In particular, the Nrf2 signaling pathway has been considered a major target of natural compounds in almost all organisms [123]. These phytochemicals are in the spotlight because they affect the expression and activity of sub-genes of Nrf2 and have been shown to regulate several diseases, including cancer [124]. This section provides information on how the phytochemicals that regulate Nrf2/KEAP1 are associated with colorectal cancer (Table 2).

### 4.1. Epigallocatechin-3-Gallate 

Epigallocatechin-3-Gallate (EGCG) is the main polyphenol in green tea [125]. EGCG has been shown to exhibit anticancer effects on various cancers through various mechanisms [126]. Moreover, EGCG has been reported to have scavenging activity of ROS and an antioxidative effect through modulation of transcription factors and enzyme activities [127]. EGCG intake (20 mg/kg) for 6 weeks has been reported to prevent colon cancer while upregulating Nrf2 and UDP-glucuronosyltransferases (UGT) genes, the phase II drug-metabolizing enzymes, in male BalB/cA nude mice [128]. In addition, EGCG intake (5, 10, 20 mg/kg) for 4 weeks in BalB/cA nude mice injected with colon cancer HT-29 cells upregulated Nrf2 protein and mRNA levels and UGT genes such as UGT1A, UGT1A8, and UGT1A10. In addition, supplementation of EGCG suppressed colon cancer growth and liver and lung metastasis and a high dose (20 mg/kg) of EGCG treatment had the best preventive effect on carcinogenesis [129]. Treatment with EGCG (12.5 μM) in colon cancer HCT-116 cells not only inhibited cell growth and colony formation, but also induced nuclear migration of Nrf2. Moreover, the EGCG intake promoted autophagy-related genes and cell death-related genes [130].

### 4.2. Sulforaphane 

Sulforaphane (SFN) is abundant in cruciferous plants and is a phytochemical of isothiocyanates [131]. SFN has antioxidant, anti-inflammatory, and anticancer properties [132] and is widely known as an activator of Nrf2, a key regulator of cellular redox balance [133]. SFN promoted Nrf2 expression and Nrf2-mediated cytoprotective response in both p53-wildtype (WT) and p53-knockout (KO) human colon cancer cells in a biphasic manner [134]. However, treatment with SFN promoted tumor growth and reduced cell death by upregulating the Nrf2 signaling pathway in a p53-dependent manner [134]. SFN increased the expression of Nrf2, which promoted tumor growth and decreased apoptotic cell death in the p53-WT xenograft animal model, whereas SFN increased apoptosis and decreased cell proliferation in the p53-KO xenograft model [134]. p53 has been reported to inhibit the expression of antioxidant enzymes by binding directly to the Nrf2-activated promoter elements [135]. Regarding these reports, the ingestion of Nrf2-activating phytochemicals including SFN could be harmful to colorectal cancer patients carrying the WT p53 gene. However, other studies have reported that SFN increases the nuclear translocation of Nrf2, inhibits cell proliferation and colony formation, and induces apoptosis in colon cancer HT-29 and SW480 cells [136].

### 4.3. Curcumin

Curcumin is extracted from the dried rhizomes of *Curcuma longa* and has long been used as a food additive and herbal medicine in Asian countries [137]. It is primarily known to have anti-inflammatory, antioxidant, and anticancer effects and has a wide range of pharmacological properties [138]. In wildtype C57BL/6J and Nrf2 knockout mice treated with single oral dose of curcumin (1,000 mg/kg), the intestine was extracted and analyzed for Nrf2-dependent genes. Among 222 detoxification enzymes (154 increased and 68 decreased), HO-1, UGT2B5, and carbonyl reductase 3 were expressed the most after curcumin treatment, followed by GST isoforms. In addition, the expression of Nrf2-dependent genes related to ubiquitination, drug metabolism, cell growth and adhesion, phosphorylation, and transcription was also increased [139].

### 4.4. Luteolin

Luteolin is a flavonoid present in honeysuckle and chrysanthemums [140]. Foods including celery, Chinese cabbage, and cauliflower contain large amounts of luteolin [141]. Luteolin is primarily known to have anti-inflammatory, antioxidant, and anticancer properties [141]. In colon cancer HT29 cells, luteolin dose-dependently exhibited cytotoxicity, and it was reported that ROS was increased due to decreased ROS scavenging ability. In addition, luteolin has been reported to increase the expression of cell death proteins such as cytochrome c, bcl-2-associated X protein (Bax), and caspase-3 by increasing the nuclear transport of Nrf2. As a result, it was found that luteolin inhibits the proliferation of HT29 cells and induces apoptosis [142]. Other studies reported that luteolin inhibits methylation of the Nrf2 promoter region in colon cancer HT-29 and SNU-407 cells, thereby increasing apoptosis-related proteins and antioxidant enzymes such as GCLC, glutathione synthetase (GSS), catalase, and HO-1 [143].

### 4.5. Allicin

Allicin (diallyl thiosulfinate) is a compound that is isolated from freshly crushed garlic [144]. It has been found primarily to have anti-inflammatory, antimicrobial, and various biological effects [145]. Allicin has been reported to induce apoptosis due to the enhancement of hypodiploid DNA content and cytochrome c release from the mitochondria to the cytoplasm, leading to decreased levels of Bcl-2 and increased levels of Bax in HCT-116 cells [146]. Allicin has also been shown to induce the nuclear translocation and promoter-binding activity of Nrf2 and knockdown of Nrf2 abrogated allicin-induced apoptosis in HCT-116 cells, demonstrating that Nrf2 mediates allicin-induced apoptotic death of colon cancer cells [146]. Allicin could interact with KEAP1, disrupting the binding of Cul3 ubiquitin ligase to KEAP1 and allowing Nrf2 to translocate to the nucleus, leading to the transcription of antioxidant genes [147]. The important role of Nrf2 in inducing apoptosis through the involvement of NF-κB signaling in colorectal cancer cells has been suggested by our group [148]. Allicin is easily metabolized into a variety of compounds, such as diallyl trisulfide, reacts with thiols and glutathione, and is easily destroyed by allinase, which makes it difficult for clinical application.

### 4.6. Resveratrol

Resveratrol (3,5,4’-trihydroxy-trans-stilbene) is a phenolic compound that is a phytochemical present in many plants, including grapes, peanuts, and berries [149]. Resveratrol has been shown to have pharmacological functions including anti-inflammatory and anticancer effects in a variety of diseases, and is known to inhibit lipid peroxidation [150]. Due to these pharmacological functions, resveratrol has been widely studied in clinical research as a functional food and a therapeutic agent for many diseases [149]. In azoxymethane (AOM)-induced colon tumorigenesis in a BalB/c mouse model, treatment with 250 ppm of resveratrol has been reported to increase the expression of Nrf2 and Nrf2-mediated antioxidative enzymes, including HO-1 and glutathione reductase [151]. The treatment with resveratrol increased cell viability and the activity of antioxidant enzymes such as SOD, CAT, GSH, and HO-1, which were suppressed by H_2_O_2_-induced intestinal barrier injury in intestinal epithelial IPEC-J2 cells [152]. Moreover, resveratrol increased the mRNA levels of Nrf2 in IPEC-J2 cells treated with H_2_O_2_ [152]. These results suggest that resveratrol can increase the expression of antioxidants through the activation of Nrf2, and that this action may contribute to the protection of intestinal cells.

### 4.7. Nobiletin

Nobiletin (5,6,7,8,3’,4’-hexamethoxyflavone) is a polymethoxylated flavone found primarily in citrus fruits [153]. Nobiletin has been reported to exhibit several biological effects, including antioxidant, anti-inflammatory, anti-obesity, and anticancer properties [154]. In a colon cancer model induced by AOM-dextran sulfate sodium (DSS) in CD-1 mice, dietary intake of 0.05% nobiletin was reported to increase the protein levels of Nrf2 and increase the translocation to the nucleus [155]. In addition, 0.05% nobiletin treatment has been reported to increase the protein levels of the antioxidant enzymes HO-1 and NQO1, which are regulated by Nrf2, and cell cycle arrest [155].

### 4.8. Genistein

Genistein, a major isoflavone in soybean-based foods, is known to have many pharmacological effects, such as tyrosine kinase inhibition, cell cycle regulation, and antioxidant and anti-inflammatory effects. In a dimethylhydrazine-induced colon cancer model in Wistar rats, genistein treatment (2.5 mg/kg body weight) for 6 weeks increased Nrf2 and HO-1 protein expression and maintained the levels of the antioxidant enzymes glutathione and NADPH at the same level as that of normal mice [156]. It has also been reported to ultimately inhibit the production of colon crypt foci [156].

### 4.9. Miscellaneous

Baicalein is a phenolic flavonoid with three hydroxyl groups derived from the *Scutellaria baicalensis* plant and is used to treat anti-inflammatory, cardiovascular, and gastrointestinal diseases [157]. Baicalein (40 μM) showed the activation of phosphorylated Nrf2 in colon cancer HCT-116 cells, and this result was reported to be due to the redox activity of baicalein [157]. In addition, treatment with baicalein decreased cell viability in a dose-dependent manner in colon cancer HT-29, HCT-116, SW480, and SW620 cells [158]. In particular, it has been reported that simultaneous treatment of chloroquine and baicalein in HCT-116 cells not only increased the autophagy marker LC3-II, but also activated caspase-3, which induces apoptosis [158].

Wogonin is extracted from the root of *Scutellaria baicalensis Georgi* and has been used primarily to treat inflammatory diseases [159]. In AOM- and DSS-induced mouse models, administration of wogonin has been shown to inhibit cancer development and promote nuclear migration of Nrf2. In addition, treatment with wogonin inhibits the levels of interleukin (IL)-6 and IL-1β in human colon cancer HCT-116 cells. Moreover, the protein levels of KEAP1 were downregulated and nuclear translocation of Nrf2 was induced [159].

Oroxylin A is a component of the root of *Scutellaria baicalensis*, a traditional Chinese herbal medicine, and is known to have cytoprotective, anti-inflammatory, and anticancer activity [160]. Treatment with oroxylin A has been reported to induce the expression of Bax protein and caspase-3 and -9 activities, while inhibiting the expression of Bcl-2 protein in HCT-116 cells [161]. Oroxylin A has also been shown to increase intracellular ROS levels to initiate signaling pathways associated with anticancer effects, such as increased nuclear translocation of Nrf2 and increased expression of antioxidant enzymes such as HO-1 and NQO1 [161]. Moreover, oroxylin A significantly reduced tumor volume and weight in the human colon cancer HCT-116 cell-injected xenograft animal model [161].

Ginnalin A is extracted from the leaves of *Acer tataricum subsp* [162]. Ginnalin A has been used as a medicinal plant in East Asia for a long time and has been reported to have antioxidant, anti-diabetic, and anticancer effects [163,164]. In a recent study, Ginnalin A treatment inhibited colony formation in colon cancer HCT-116, SW480, and SW620 cells and reduced cell proliferation by inducing cell cycle arrest at S-phase. In addition, treatment with Ginnalin A increased the mRNA expression of the Nrf2-related antioxidant genes Nrf2, HO-1, and NQO1. It was also found to increase the nuclear translocation of Nrf2, which not only increased the protein expression of Nrf2, HO-1, and NQO1, but also inhibited KEAP1, a negative regulator of Nrf2 [162].

**Table 2:** List of phytochemicals targeting Nrf2/KEAP1 for colorectal cancer.

Phytochemicals	Effective Dose (Periods)	Experimental Model	Mode of Action	Reference
EGCG	20 mg/kg (6 weeks)	BalB/cA nude mouse	Nrf2 mRNA levels ↑ UGT mRNA levels ↑ Phase II drug metabolizing enzymes ↑	[128]
	5, 10, 20 mg/kg (4 weeks)	BalB/cA nude mouse	Nrf2 protein levels ↑ Nrf2 mRNA levels ↑ UGT1A, UGT1A8 and UGT1A10 mRNA levels ↑ Liver and lung metastasis (20 mg/kg) ↓	[129]
	12.5 μM	HCT-116 cell	Cell growth ↓ Colony formation ↓ Nrf2 nuclear translocation ↑ LC3B protein levels ↑ Caspase-9 protein levels ↑	[130]
SFN	0, 1, 10 μM	HCT-116	Nrf2 protein levels ↑ HO-1 protein levels ↑ mtDNA/nDNA ratio (p53 KO) ↓ PGC-1α (p53 KO) ↓	[134]
	2.5, 10, 25 mg/kg	p53 wildtype (WT) and p53 knockout (KO) BalB/c nude mouse	Nrf2 protein levels (p53 KO) ↑ Bcl-2 protein levels (p53 KO) ↓ Cytochrome C (p53 KO) ↑	[134]
	5, 10, 15, 20 μM	HT29 and SW480 cell	Nrf2 protein levels ↑ UGT1A protein levels ↑ Cell proliferation ↓ Cell migration ↓ Colony formation ↓ Apoptosis ↑	[136]
Curcumin	1000 mg/kg (single oral dose)	Nrf2 WT and Nrf2 KO C57BL/6J mouse	HO-1 mRNA levels ↑ UGT2B5 mRNA levels ↑ Carbonyl reductase 3 ↑	[139]
Luteolin	20, 40 μM	HT29 cell	ROS ↑ Cytochrome C protein levels ↑ Bax protein levels ↑ Bcl-2 protein levels ↓ Caspase-3 protein levels ↑ Nrf2 nuclear translocation ↑ Cell proliferation ↓	[142]
	10, 30, 60 μM	HT29 and SNU-407 cell	Methylation of Nrf2 promoter ↓ GCLC protein levels ↑ GSS protein levels ↑ Catalase protein levels ↑ HO-1 protein levels ↑ Bax protein levels ↑ Bcl-2 protein levels ↓ Cleaved caspase-3 protein levels ↑ Cleaved caspase-9 protein levels ↑ Cell viability ↓	[143]
Allicin	10 μg/ml	HCT-116 cell	Nrf2 protein levels ↑ Nrf2 promoter activity ↑ Cytochrome C ↑ Bax protein levels ↑ Bcl-2 protein levels ↓	[146]
Resveratrol	250 ppm	BalB/c mouse (AOM model)	Nrf2 protein levels ↑ HO-1 protein levels ↑ Glutathione reductase mRNA levels ↑	[151]
	20, 50 μM	IPEC-J2 cell (H_2_O_2_-induced intestinal barrier injury)	Cell viability ↑ SOD activity ↑ CAT activity ↑ GSH activity ↑ SOD mRNA levels ↑ CAT mRNA levels ↑ HO-1 mRNA levels ↑ Nrf2 mRNA levels ↑	[152]
Nobiletin	AIN93G diet containing 0.05% nobiletin	CD-1 mouse (AOM/DSS model)	Nrf2 nuclear translocation ↑ Nrf2 protein levels ↑ HO-1 protein levels ↑ NQO-1 protein levels ↑ Cell cycle arrest	[155]
Genistein	2.5 mg/kg body weight (6 weeks)	Wistar rat (dimethylhydrazine-induced colon carcinogenesis)	Nrf2 expression ↑ HO-1 expression ↑ Glutathione levels ↑ NADPH levels ↑	[156]
Baicalein	40 μM	HCT-116 cell	Nrf2 phosphorylation ↓	[157]
	50, 100, 150, 200 μM	HT29, SW480, HCT-116, and SW620 cell	Cell viability ↓ LC3-II protein levels ↑ (HCT-116) Caspase-3/7 activity ↑ (HCT-116)	[158]
Wogonin	60 mg/kg (29, 48, 68, 105 days)	C57BL/6 mouse (AOM/DSS model)	Nrf2 nuclear translocation ↑ Nrf2 positive cells ↑	[160]
	25, 50, 100 μM	HCT-116 cell	IL-6, IL-6, IL-1β levels ↓ HO-1 protein levels ↑ NQO1 protein levels ↑ KEAP1 protein levels ↑ Nrf2 promoter activity ↑	
Oroxylin A	50, 100, 150 μM	HCT-116 cell	Cell proliferation ↓ Bax protein levels ↑ Bcl-2 protein levels ↓ Nrf2 nuclear translocation ↑ HO-1 protein levels ↑ NQO1 protein levels ↑	[161]
	50, 100, 200 mg/kg	Balb/c mouse	Nrf2 nuclear translocation ↑	
Ginnalin A	20, 40, 80 μM	HCT-116, SW480, and SW620 cell	Cell proliferation ↓ S-phase cell cycle arrest Nrf2 mRNA and protein levels ↑ HO-1 mRNA and protein levels ↑ NQO1 mRNA and protein levels ↑ KEAP1 protein levels ↓	[162]

EGCG; epigallocatechin-3-gallate, SFN; sulforaphane, Nrf2; nuclear factor erythroid 2-related factor 2, KEAP1; kelch-like ECH-related protein 1, AOM; azoxymethane, UGT; glucuronosyltransferases, mtDNA; mitochondrial DNA, nDNA; nuclear DNA, PGC-1α; peroxisome proliferator-activated receptor-gamma coactivator-1 alpha, HO-1; heme oxygenase-1, ROS; reactive oxygen species, Bcl-2; B-cell lymphoma 2, Bax; Bcl-2-associated X protein, GCLC; glutamate-cysteine ligase catalytic subunit, GSS; glutathione synthetase, SOD; superoxide dismutase, CAT; catalase, GSH; glutathione, NQO-1; NAD(P)H quinone oxidoreductase 1.

## 5. Clinical Trials of Phytochemicals in Patients with Colorectal Cancer

As mentioned in the previous section, many phytochemicals have been demonstrated in vitro and in vivo studies to inhibit CRC progression or induce apoptosis of CRC cells. This section introduces clinical trials of phytochemicals in CRC. After surgery, patients with phase I and II CRC within 4 to 12 weeks were given 450 mg of EGCG twice a day for 1 year, and there was a clinical trial confirming chemoprevention, but it has not yet been fully identified, and further investigation is required (NCT02891538). In a clinical trial for curcumin, patients with CRC phase I were given 4 g of curcumin once a day, and phase II patients with CRC were given FOLFOX (Folinic acid, Fluorouracil, and Oxaliplatin) with curcumin, followed by a clinical trial confirming chemoprevention. However, this has not yet been fully disclosed (NCT01490996). In another clinical trial for curcumin in patients with phase II CRC, 2 g or 4 g of curcumin was ingested for 30 days, followed by aberrant crypt foci (ACF), and it found that daily intake of 4 g of curcumin could reduce ACF counts [165]. In a clinical trial for resveratrol, an experiment was conducted to test the hypothesis that resveratrol regulates the Wnt signaling pathway. Patients with phase I CRC were given 20 or 80 g of resveratrol per day, and as a result, it was reported that the expression of Wnt target genes was suppressed (NCT00256334) [166]. In a clinical trial for genistein, patients who took genistein 60 mg daily for 2 weeks and those who took genistein with FOLFOX or FOLFOX-Bevacizumab were tested. As a result, it was reported that no increased side effects were observed when genistein was taken with FOLFOX or FOLFOX-Bevacizumab (NCT01985763 [167]. As listed, clinical studies of EGCG, curcumin, resveratrol, and genistein have been reported (Table 3), but in CRC, clinical studies have been conducted with only a limited number of phytochemicals and there are no studies related to ROS scavenging and the Nrf2/KEAP1 pathway. Clinical trials should also be supported, as the beneficial effects of phytochemicals in CRC are scientifically proven.

**Table 3:** Clinical trials on phytochemicals in patients with colorectal cancer.

Phytochemicals	Dosage (Periods)	Phase	Results	NCT Number	Reference
EGCG	900 mg (1 year)	I, II	Chemoprevention	NCT02891538	-
Curcumin	4 g	I	Chemoprevention	NCT01490996	-
	2, 4 g (30 days)	II	Aberrant crypt foci ↓	-	[165]
Resveratrol	20, 80 g	I	Wnt signaling pathway regulation	NCT00256334	[166]
Genistein	60 mg (2 weeks)		No observation of side effects with/without FOLFOX or FOLFOX-Bevacizumab	NCT01985763	[167]

## 6. Conclusions

**Figure 6:**
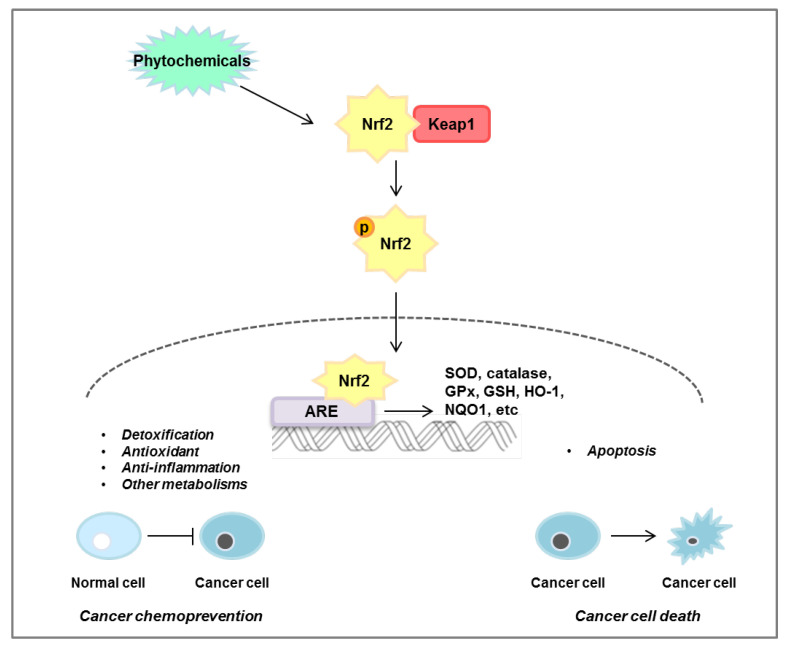
Mechanisms of Nrf2/KEAP1 pathway regulated by phytochemicals. Phytochemicals induce nuclear translocation of Nrf2, which then binds to the ARE in the nucleus, leading to the expression of siperoxide dismutase (SOD), catalase, glutathione peroxidase (GPx), glytathione (GSH), heme oxygenase-1 (HO-1), and NAD(P)H quinone oxidoreductase 1 (NQO1). This pathway plays an important role in cancer chemoprevention by regulating detoxification, antioxidants, anti-inflammation, and other metabolisms in normal cells. Conversely, phytochemicals induce apoptosis in cancer cells through regulation of the Nrf2/KEAP1 pathway.

Over time, it has been shown that ROS are associated with many diseases. ROS-induced lipid peroxidation products, such as 4-hydroxynonenal, acrolein, malondialdehyde, and hexanal, have a long half-life and can act as secondary messengers of oxidative stress, which can play an important role in promoting various diseases. In particular, unlike ROS which have a short half-life, by-products of lipid peroxidation, which have a fast diffusion rate and a long half-life, can cause secondary stress in cells. The Nrf2/KEAP1 signaling pathway plays a dual role in normal and cancer cells. As shown in Figure 6, activation of the Nrf2/KEAP1 signaling pathway leads to detoxification and antioxidant and anti-inflammatory effects in normal cells, but induces apoptosis in cancer cells and induces the death of cancer cells. Research targeting Nrf2 to treat or prevent cancer is ongoing, but the mechanisms in the body following dietary intake have not been fully elucidated. However, it has been clearly found that the intake of phytochemicals has a preventive or inhibitory effect depending on the stage of colorectal cancer. In accordance with this, numerous studies are also ongoing to find effective phytochemicals on the Nrf2/KEAP1 axis and discover the efficacy of their derivatives. In this review, we have demonstrated the significance of the Nrf2/KEAP1 signaling pathway and the anticancer effect of phytochemicals targeting the Nrf2/KEAP1 axis in colorectal cancer cells as well as animal models (Table 2). However, validation through clinical research and studies on phytochemicals that control colorectal cancer through the Nrf2/KEAP1 signaling pathway are still insufficient and further research is required.

## Abbreviations

Acr-dG	Acrolein-induced DNA damages
APC	Adenomatous polyposis coli
ARE/EpRE	Antioxidant/electrophile responsive element
BTB	Broad complex/Tramtrack/Bric-a-brac
bZIP	Basic leucine zipper
CAC	Colitis-associated colorectal cancer
CBP	CREB-binding protein
CD	Crohn’s disease
CHD6	Chromo-ATPase/helicase DNA-binding protein 6
CNC	Cap ’N’ Collar
CRC	Colorectal cancer
CREB	cAMP response element binding protein
Cul3	Cullin 3
DGR	Double glycine-repeat
EGCG	Epigallocatechin-3-Gallate
EGFR	Epithelial growth factor receptor
ERK	Extracellular-signal-regulated kinase
GCLC	Glutamate-cysteine ligase catalytic subunit
GCLM	Glutamate-cysteine ligase modifier subunit
GPx	Glutathione peroxidase
GSH	Glutathione
GSK	Glycogen synthase kinase
GST	Glutathione-S-transferase
HO	Heme oxygenase
IBD	Inflammatory bowel disease
IVR	Intervening region
KEAP1	Kelch-like-ECH-associated protein 1
NADPH	Nicotinamide adenine dinucleotide phosphate
Neh	Nrf2-ECH homology
Nrf2	Nuclear factor erythroid 2-related factor 2
NQO1	NAD(P)H quinone oxidoreductase 1
PTEN	Phosphatase and tensin homolog
PUFA	Polyunsaturated fatty acids
ROS	Reactive oxygen species
SFN	Sulforaphane
sMAF	Small musculoaponeurotic fibrosarcoma protein
SOD	Superoxide dismutase
TGF-βRI	Transforming growth factor-beta receptor I
β-TrCP	β-transducin repeat-containing protein
UbcM2	Ubiquitin-conjugating enzyme
UC	Ulcerative colitis
UGT	UDP-glucuronosyltransferases
